# Comparison of the outcomes between total arch replacement and nontotal arch replacement in patients with acute type A aortic dissection

**DOI:** 10.1007/s12055-023-01576-7

**Published:** 2023-08-15

**Authors:** Pichej Lerdpunnapongse, Worawong Slisatkorn, Wanchai Wongkornrat, Vutthipong Sanphasitvong

**Affiliations:** 1 Cardiovascular and Thoracic Surgery Unit, Heart Center, Chulabhorn Hospital, Bangkok, Thailand; 2 Division of Cardio-Thoracic Surgery, Department of Surgery, Faculty of Medicine Siriraj Hospital, Mahidol University, 2 Wanglang Road, Bangkoknoi, Bangkok, 10700 Thailand

**Keywords:** Aortic arch, Acute aortic dissection, Aortic dissection type A, Total arch replacement

## Abstract

**Objective:**

To compare the outcomes between total arch replacement (TAR) and nontotal arch replacement (non-TAR) in patients with acute type A aortic dissection (ATAAD).

**Methods:**

Between 2006 and 2018, 275 ATAAD patients were divided into 2 groups, the TAR group (*n* = 63) and the non-TAR group (*n* = 212), and multiple variables were analyzed.

**Results:**

The TAR patients were older than the non-TAR patients (61.5 ± 11.8 vs. 57.4 ± 14.5 years, *p* = 0.024). The TAR group had longer operative, cardiopulmonary bypass, aortic clamping, and circulatory arrest times than the non-TAR group (all *p* < 0.001). The overall hospital mortality rate was 8.7% with no statistically significant difference between the TAR and non-TAR groups (9.5% vs. 8.5%, *p* = 0.799). There was no significant difference in the incidence of acute kidney injury (AKI), intubation time, incidence of postoperative atrial fibrillation (AF), or reoperation for bleeding or reintervention rates between the TAR and non-TAR groups (68.3% vs. 65.7% (*p* = 0.912), 44.8% vs. 33.8% (*p* = 0.127), 30.2% vs. 22.6% (*p* = 0.222), 9.5% vs. 9.5% (*p* = 0.189), and 7.9% vs. 7.1% (*p* = 0.077), respectively). The TAR group had a higher rate of new permanent neurological deficit (PND) than the non-TAR group and longer median length of hospital stay (17.5% vs. 6.1% (*p* < 0.001) and 9 vs. 12 days (*p* = 0.049), respectively). TAR (relative risk (RR) 3.66, *p* = 0.005) and preoperative cardiopulmonary resuscitation (CPR) (RR 6.60, *p* = 0.019) were risk factors of PND. Survival rate was similar between the two groups.

**Conclusion:**

The mortality rates in ATAAD patients with TAR and non-TAR were similar. However, the incidence of new permanent postoperative neurological deficit was significantly higher, and the length of hospital stay was longer in patients with TAR. TAR in ATAAD should be avoided especially in patients who have experienced preoperative CPR to abate risk of PND.

## Background

Acute type A aortic dissection (ATAAD) is a life-threatening medical emergency that requires prompt surgical intervention due to its early mortality rate of up to 1% per hour in the first 48 h, if left untreated [1, 2]. The choice of surgical approach depends on the extent of aortic dissection, the presence of associated comorbidities, and the surgeon’s preference, so there have been numerous advances in surgical techniques, especially in distal reconstruction [3–6]. In total arch replacement (TAR), the entire aortic arch is replaced with a prosthetic graft. In nontotal arch replacement (non-TAR), only a portion of the aortic arch is replaced, limiting the procedure to ascending aortic replacement, hemiarch replacement, and partial arch replacement (arch replacement with distal anastomosis at zone 1 or 2). The optimal extent of distal reconstruction remains uncertain. Overall, TAR is associated with a longer cardiopulmonary bypass time, a longer circulatory arrest time, a higher risk of postoperative stroke, and a higher mortality rate [7]. On the other hand, non-TAR may result in dilatation of the residual dissected aorta, and reintervention in the future may be needed [8–10]. To provide further data to help clarify this still unsolved debate, we designed this retrospective single-center study to compare the outcomes between TAR and non-TAR.

## Patients and methods

### Study population

From January 2006 to December 2018, a total of 275 patients with ATAAD underwent surgery in our center. These patients were divided into 2 groups, with 63 (23%) in the TAR group and 212 (77%) in the non-TAR group. All patients who underwent surgery for ATAAD were included in the study. Patients who had previously undergone a cardiac operation or were under 18 years old were excluded from the study. The primary end point was hospital mortality, which was defined as death in the hospital within 30 days after surgery. The secondary end points were as follows: acute kidney injury (AKI), as defined by an increase in the serum creatinine level of 2 mg/dl or more; prolonged intubation, as defined by the inability to be extubated within 48 h; new permanent neurological deficit (PND), as defined by a new evident permanent brain injury found via clinical and/or radiological examination; postoperative atrial fibrillation (AF); reoperation for bleeding and reintervention. This study was reviewed and ethically approved by the Siriraj Institutional Review Board for Human Research as per the Declaration of Helsinki, International Conference on Harmonization Good Clinical Practice (ICH-GCP), and Council for International Organizations of Medical Sciences (CIOMS) guidelines.

### Surgical technique

Our surgical technique has been described in detail. To establish a heart–lung machine, we typically drain from the right atrium and inflow to the right axillary artery and/or femoral artery. The patient’s core body temperature is lowered to approximately 20–25 °C during the surgical procedure. We temporarily stop the blood flow through the aorta and reroute the cerebral blood flow via selective antegrade cerebral perfusion (SACP) at a rate of 10–15 ml/kg/min. Distal anastomosis of the TAR is routinely performed in zone 3 after removing the pathological aorta. We usually use a four-branch prosthetic graft to construct the new aortic arch. Additionally, bifrontal near-infrared spectroscopy (NIRS) is routinely used to monitor cerebral oxygenation. This meticulous approach ensures that our surgical techniques are optimized for the highest level of success and patient safety.

### Data analysis

Statistical analyses were performed by using SPSS 20 for Macintosh. Categorical variables were compared using 2-sided chi-square analysis or Fisher’s exact test where appropriate. Continuous variables were compared using independent-sample parametric (unpaired Student’s *t*) or nonparametric (Mann–Whitney *U*) tests.

Variables with univariable *p* values < 0.2 or those of interest were candidates in multivariable analysis using forward stepwise binary logistic regression. The Kaplan–Meier curve was applied for survival outcome. A *p* value < 0.05 was considered statistically significant.

## Results

**Fig. 1:**
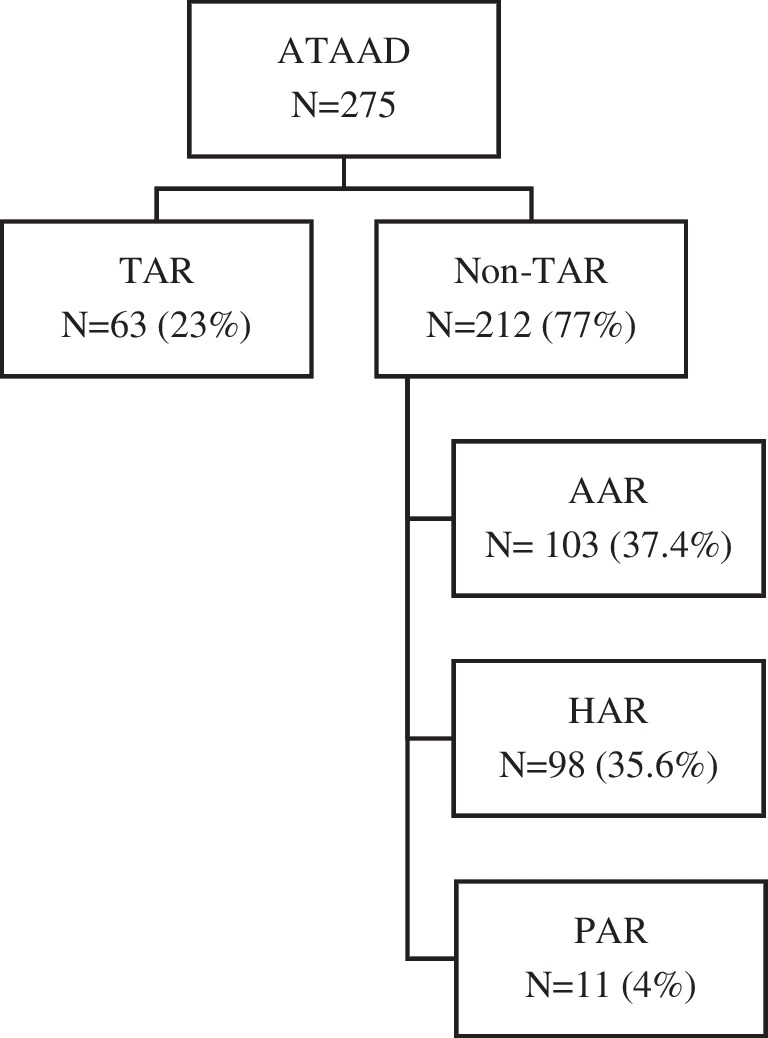
Diagram demonstrating operative management of ATAAD

Between January 2006 and December 2018, 275 patients who underwent surgical repair for ATAAD were identified. Sixty-three patients were in the TAR group, and 212 patients were in the non-TAR group, including 103 with ascending aortic replacement (AAR), 98 with hemiarch replacement (HAR), and 11 with partial arch replacement (PAR) (Fig. 1).

### Patient demographic characteristics

There was no significant difference in sex, hypertension, or body mass index between the two groups. The patients in the TAR group were significantly older than those in the non-TAR group (mean age of 61.5 vs. 57.4 years old, *p* = 0.024). The proportion of patients with Marfan syndrome or a familial history of aortic disease or bicuspid aortic valve (BAV) was low and was not different between the two groups. The rates of preoperative critical conditions, including cardiac tamponade, shock, intubation, and cardiopulmonary resuscitation (CPR), were similar. The patient demographic characteristics are shown in Table 1.

**Table 1:** Patient demographic characteristics

Characteristic	Non-TAR	TAR	*p* value
*N*	212	63	
Male	132 (62.3%)	40 (63.5%)	0.86
Age (mean ± SD, years)	57.4 ± 14.5	61.5 ± 11.8	0.024
BMI (mean ± SD, kg/m^2^)	24.8 ± 4.4	24.7 ± 3.7	0.86
Hypertension	165 (80.1%)	49 (77.8%)	0.96
Marfan syndrome	9 (4.2%)	1 (1.6%)	
Family history	7 (3.3%)	1 (1.8%)	0.684
BAV	6 (2.8%)	0	0.342
Intubation	23 (10.8%)	6 (9.5%)	0.764
Tamponade, shock	34 (16%)	7 (11.1%)	0.335
CPR	8 (3.8%)	1 (1.6%)	0.689

*SD*, standard deviation; *BMI*, body mass index

### Intraoperative details

There was no difference in the sites or techniques of arterial or venous cannulation. The concomitant procedures, such as aortic root surgery, coronary artery bypass, and valvular surgery, were similar between the two groups. The TAR group had significantly longer operative (median, 480 vs. 350 minutes (min), *p* < 0.001), cardiopulmonary bypass (median, 211 vs. 149 min, *p* < 0.001), aortic cross-clamp (median, 148 vs. 110 min, *p* < 0.001), and circulatory arrest times (median, 70 vs. 38 min, *p* < 0.001). The lowest temperature was significantly lower in the TAR group (median, 22 °C vs. 24 °C, *p* = 0.02). Nineteen patients underwent TAR with the frozen elephant trunk technique (19/63, 30.2%). Intraoperative details are summarized in Table 2.

**Table 2:** Intraoperative details

Variables	Non-TAR	TAR	*p* value
Arterial cannulation			0.347
Ascending aorta	22 (10.4%)	4 (6.3%)	
Innominate artery	18 (8.5%)	5 (7.9%)	
Right axillary artery	91 (43.1%)	35 (55.6%)	
Femoral artery	80 (37.9%)	19 (30.2%)	
Venous cannulation			0.715
Right atrium	173 (81.6%)	50 (79.4%)	
Femoral vein	39 (18.4%)	13 (20.6%)	
Concomitant procedure			
Aortic root surgery	33 (15.6%)	5 (7.9%)	0.123
CABG	17 (8%)	8 (12.7%)	0.257
Valve surgery	12 (5.7%)	4 (6.3%)	0.766
	Median (P25, P75)	Median (P25, P75)	
Operative time (min)	350 (300, 431)	480 (385, 612)	< 0.001
CPB time (min)	149 (120, 203)	211 (170, 277)	< 0.001
Aortic clamping time (min)	110 (80, 148)	148 (125, 244)	< 0.001
Circulatory arrest time (min)	38 (27, 54)	70 (51, 106)	< 0.001
Temperature (°C)	24 (21, 25)	22 (20, 24)	0.002

*CABG*, coronary artery bypass grafting; *CPB*, cardiopulmonary bypass

### Postoperative outcomes

The mortality rate was similar between the two groups (TAR 9.5% vs. non-TAR 8.5%, *p* = 0.799).

The incidence of new PNDs was significantly higher in the TAR group (17.5% vs. 6.1%, *p* < 0.001). The major complications were AKI, prolonged intubation > 48 h, postoperative AF, reoperation from bleeding, and reintervention, with no significant differences between the two groups. The length of intensive care unit (ICU) stay was similar between two groups, but the length of hospital stay was significantly longer in the TAR group. Postoperative outcomes are shown in Table 3.

**Table 3:** Postoperative outcomes

Variables	Non-TAR	TAR	Unadjust	Adjust*	
			*p* value	RR (95%CI)	*p* value
Mortality	18 (8.5%)	6 (9.5%)	0.799		
Survival time (months), mean ± SD	35.3 ± 40.9	24.6 ± 27.9	0.761		
AKI	139 (65.7%)	43 (68.3%)	0.912	1.01 (0.83, 1.22)	0.958
AKI with dialysis	34 (16%)	10 (15.9%)			
Prolonged intubation > 48 h	66 (33.8%)	26 (44.8%)	0.127	1.34 (0.93, 1.91)	0.114
Postoperative AF	48 (22.6%)	19 (30.2%)	0.222	1.20 (0.77, 1.89)	0.423
New permanent neurological deficit (PND)	13 (6.1%)	11 (17.5%)	< 0.001	2.64 (1.24, 5.65)	0.012
Reoperation due to bleeding	20 (9.5%)	6 (9.5%)	0.189	0.99 (0.41, 2.36)	0.973
Reintervention	15 (7.1%)	5 (7.9%)	0.786	1.18 (0.44, 3.17)	0.748
Aortic related	14 (6.6%)	2 (3.2%)	0.539		
Length of ICU stay (days), median (P25, P75)	3 (2–6)	4 (3–8)	0.078		
Length of hospital stay (days), median (P25, P75)	9 (7–16)	12 (8–20)	0.049		

^*^Adjust, adjusted for age

*RR*, relative risk; *CI*, confidence interval

**Fig. 2:**
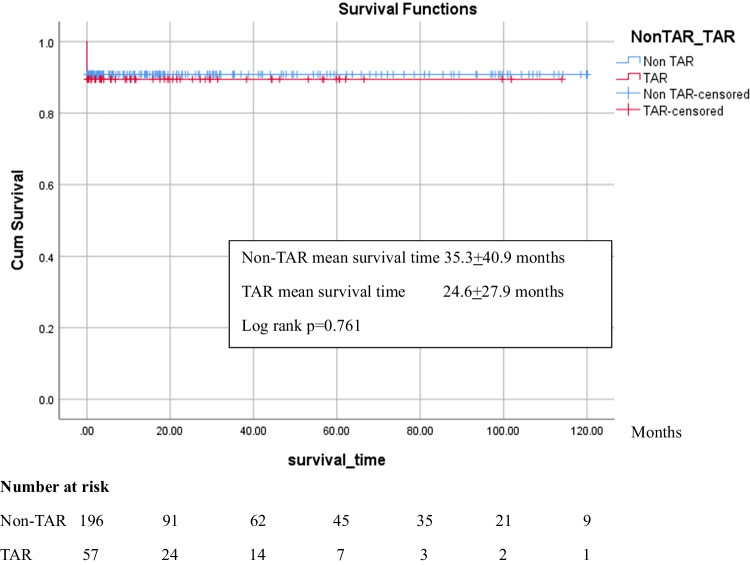
Kaplan–Meier curve demonstrating survival after operation

To analyze if TAR was the risk factor for PND, we performed univariate/multivariate analysis of potential risk factors as shown in Table 4. TAR (RR 3.67, *p* = 0.004) and preoperative CPR (RR 7.77, *p* = 0.008) were risk factors of PND. There was no significant difference in survival after operation between two groups. The Kaplan–Meier curve of survival is demonstrated in Fig. 2.

**Table 4:** Risk factors for permanent neurological deficit

Variables	Univariate analysis		Multivariate analysis	
	OR (95% CI)	*p* value	Adjusted OR (95% CI)	*p* value
Age (years, 10 units)	1.33 (0.97–1.82)	0.073		
Arterial cannulation	1.46 (0.61–3.45)	0.392		
TAR	3.24 (1.37–7.65)	0.005	3.67 (1.51–8.93)	0.004
Tamponade, shock	2.06 (0.76–5.54)	0.144		
CPR	5.83 (1.36–25.01)	0.035	7.77 (1.70–35.42)	0.008

## Discussion

In this study, we present our 13-year experience in managing ATAAD with TAR or non-TAR. The strategy to manage distal anastomosis depends on many factors, such as intimal tears in the aortic arch, aortic arch diameter, tears in the proximal descending aorta, and the status of the true and false lumen downstream. If an intimal tear is found in the ascending aorta, AAR or HAR will be performed. However, when the intimal tear is close to the origin of supra-aortic vessels, partial aortic arch replacement or TAR will be options to eliminate the pathological segment. TAR with frozen elephant trunk is used in cases of intimal tears in the proximal descending aorta or small true lumen diameter [11]. The goal of management is to save the patient’s life in emergency conditions; so in complex situations, the selected procedure may not be as extensive as in the strategy previously mentioned.

First, we found that patients with ATAAD had an overall hospital mortality rate of 8.7%, which was acceptable compared to 18% in the International Registry of Acute Aortic Dissection (IRAD), 16.9% in the German Registry, and 11.8% in the Japanese Registry [12–14]. There was no statistically significant difference in mortality between TAR and non-TAR patients. This may be due to similarities in preoperative patient characteristics and preoperative shock or cardiac tamponade. Our result is similar to that in the systematic review by Poon et al. [7]. Second, while the type of distal reconstruction was expected to correlate with PND, our findings showed no correlation with other morbidities, including AKI, prolonged intubation time, postoperative AF, or reoperation for bleeding. As expected, patients who underwent TAR required a longer stay in the ICU and hospital than non-TAR patients, but the difference was not statistically significant. Last, it is commonly believed that non-TAR is associated with poor false lumen remodeling and a high long-term reintervention rate. However, our study revealed no statistically significant differences in the reintervention rate between TAR and non-TAR patients.

In our study, the incidence of PND in patients who underwent TAR (17.5%) was significantly higher than in those who did not undergo TAR (6.1%), *p* < 0.001 by unadjusted and *p* = 0.012 after adjusted for age, which was different from that reported in a systematic review by Poon et al. [7]. They reported a similar incidence rate of PNDs between patients with HAR and TAR. Despite the use of SACP, NIRS and moderate hypothermia can reduce the brain’s metabolism and protect it from ischemic injury, but PNDs after TAR remain a major concern. This is because clamping time is usually longer during TAR, as the entire arch needs to be replaced. Additionally, there is also a risk that tiny particles of debris or aortic plaques may dislodge and cause embolism in the brain. Furthermore, separate reimplantation of the supra-aortic branches increases the risk of PND when compared to en bloc reimplantation [15].

Not only TAR operation, we also found that preoperative CPR is the risk factor for PND. This undesired condition was associated with poor postoperative neurological outcomes due to low preoperative cerebral blood flow and further brain ischemia during the circulatory arrest period. Furthermore, in patients with PND, the mortality rate was as high as 45.5% (5/11). Nevertheless, we remain optimistic about further research and development of more effective techniques to minimize the incidence of PND during TAR.

### Limitations

Although our study yielded valuable insights, it is important to acknowledge its limitations. First, as a single-center retrospective study, the results could have been influenced by bias and confounding factors. Second, differences in the surgeons’ skill levels should be considered as they are difficult to differentiate and may have a significant impact on clinical outcomes.

## Conclusions

The mortality rates of patients with ATAAD with TAR are similar to those with non-TAR. However, the incidence of new permanent postoperative neurological deficits was significantly higher, and the length of hospital stay was longer in patients who underwent TAR. TAR in ATAAD should be avoided especially in patients who have experienced preoperative CPR to abate risk of PND.

## Data Availability

Not applicable.
